# The Feeding of an Army

**Published:** 1899-12-09

**Authors:** 


### The Hospital

A Journal of

The Medical Sciences and Hospital Administration.

Vol. XXVII.—No. 689.] Edited by Sir Henry Burdett, K.C.B., and Solomon C. Smith, M.D., M.R.C.P. [December 9, 1899.

### THE FEEDING OF AN ARMY.

The enormous magnitude of the enterprise of feeding an army at a distance from its base of operations is, perhaps, scarcely recognised by the great majority of people; and yet, when carefully considered, it will probably be found to supply an ample explanation of the time which has been occupied in the mere preparation of our forces for a methodical advance upon the positions held by the Boers in South Africa. Even as it is, we hear that the division under Lord Methuen has been compelled to go into action at daybreak without breakfast, and to be engaged with a very formidable enemy for a period of ten hours before any meal could be supplied to the men; and it is evident that such a combination of fasting and of exertion must cast a very heavy strain upon the staying powers even of the strongest. It will evidently be the right policy so to order matters that our gallant soldiers shall not in the future be called upon to undertake arduous duty, in the way either of fighting or marching, without a plentiful supply of proper sustenance; and, for this purpose, it may now and again be necessary to submit to delays which everybody concerned would be glad to avoid. The chief compensation for them is in the obvious fact that the element of time must fight definitely on the side of the British; and that, while all the difficulties in the way of the advance of our troops will day by day be steadily surmounted, the similar difficulties which must interfere with the maintenance in efficiency of the Boer forces in the field must be as steadily increased. Their means of transport and their supplies of food and of ammunition, however considerable, must be limited, and must, therefore, be tending towards exhaustion; while ours, in consequence of our command of the sea and of a convenient base of operations, are practically inexhaustible. In these considerations we may find a full justification of the dignified patience with which Great Britain is waiting the course of events, and is submitting without a murmur to the delays which her generals find to be inevitable, as well as to the suspense which is pressing so heavily upon innumerable families and homes. We have to contend against an organisation to which years of time and millions of money have been patiently devoted, and we all feel and realise that it is necessary for us to be patient and to organise in our turn. We have learnt of late years that the health, which is so important a factor in the power and position of a State, is a still more important factor in the efficiency of an army; and that to send our troops into the field without adequate provision for their nourishment would be to expose them unnecessarily to potent causes of disease, and to curtail, without excuse, their powers of recovering from injury.

A highly interesting article in the *Times* of November 30th tells us that the provisioning of the Army rests upon the principle of maintaining a four months' supply of food at the base of operations; and that therefore, when this supply is established, it must still be replenished at short intervals, and to a sufficient extent to keep pace with the daily rate of consumption. We have to maintain in South Africa, roughly speaking, 116,000 men, including soldiers and those engaged in the work of transport, and 51,000 horses and mules. At the present time there are only supplies for three months in the country, but the necessary additions are being rapidly and continuously despatched, so that the theoretical standard of quantities will soon be attained. What these quantities are can only be stated in figures which, like those expressive of astronomical distances, practically elude the imagination. Among the chief items are twelve million pounds of preserved meat, and twelve million pounds of biscuit. At an approximate computation, the preserved meat alone would occupy a space about equal to that of a terrace of ten houses, each house thirty feet high, thirty feet deep, and with thirty feet of frontage. Then come 400,000 lbs. of coffee, 200,000 lbs. of tea, 2,200,000 lbs. of sugar, 800,000 lbs. of compressed vegetables, 400,000 lbs. of salt, and 1,500,000 lbs. of jam, as essential requirements of the military storeroom. Sugar is now universally admitted to be an admirably nutritious article of diet; and the jam affords, in combination with sugar, vegetable elements which are of high importance for the prevention of scurvy. A further provision in this direction is that of 400,000 lbs. of limejuice, while other needs in the way of liquids are met by 80,000 gallons of rum, 12,000 bottles of whisky, 32,000 bottles of port wine, a "vast quantity" of sparklets for making soda water, and 80 tons of alum for purifying spring or river water the quality of which may be doubtful. In addition to all this there is the forage for animals, and the supply, not only of these animals themselves, but also of the wagons to which they are to be harnessed, and of the harness itself by which this operation is to be effected. The first idea was to obtain wagons and mule harness in large quantities from the United States, but it was considered that they approached too nearly to "munitions of war" for this course to be pursued, and the demands are in course of being fully met by the unaided resources of the Empire.

